# The 12 Item Social and Economic Conservatism Scale (SECS)

**DOI:** 10.1371/journal.pone.0082131

**Published:** 2013-12-11

**Authors:** Jim A. C. Everett

**Affiliations:** Department of Experimental Psychology, University of Oxford, Oxford, United Kingdom; Institutes for Behavior Resources and Johns Hopkins Univ, United States of America

## Abstract

Recent years have seen a surge in psychological research on the relationship between political ideology (particularly conservatism) and cognition, affect, behaviour, and even biology. Despite this flurry of investigation, however, there is as yet no accepted, validated, and widely used multi-item scale of conservatism that is concise, that is modern in its conceptualisation, and that includes both social and economic conservatism subscales. In this paper the 12-Item Social and Economic Conservatism Scale (SECS) is proposed and validated to help fill this gap. The SECS is suggested to be an important and useful tool for researchers working in political psychology.

## Introduction

In the last few decades, there has been a proliferation of research in political psychology, and there are now entire journals dedicated to disseminating research at the interface of political science and psychology. Researchers in numerous areas of psychology and cognitive science now study the ways in which political ideology is related to other aspects of human cognition, behaviour, and biology. For example, contemporary research explores the relationship between political ideology and moral intuitions [1], genotypic differences associated with endorsement of particular ideologies [2], [3], associations between political conservatism and regional brain volume of the right amygdala [4], associations between political liberalism and conflict-related anterior cingulate activity [5], individual differences associated with support of different ideologies [6], the relationship of political ideology to belief in free will [7], and even ideological differences associated with categorization in perceptually ambiguous social groups [8] – to name just a few. While the psychological investigation of ideology (and in particular *conservatism*) is undoubtedly an important area of research, its basic integrity depends upon having an appropriate measurement of political beliefs. In this article numerous problems with current measurements of conservatism are discussed, before a new 12-item scale to measure conservatism is proposed: the 12-item Social and Economic Conservatism Scale (SECS).

### Conservatism

The left-right, or liberal-conservative, dimension has now been the primary method of classifying political ideological values for over two hundred years. In work by psychologists and political scientists, left or right wing identification has been shown to predict voting behavior [9] and shows remarkable consistency with general positions on issues such as nationalism, equality, and system maintenance ([10, 11] - p.213–214). Moreover, recent years have seen a marked increase in research suggesting that there may be consistent differences in the way liberals and conservatives think and perceive, and that these underlying differences may actually nudge individuals toward one end of the political spectrum or the other. In particular, needs for order, structure, closure, certainty, dogmatism, and discipline are often shown to be more central to the thinking of conservative proponents [12], whereas higher tolerance of ambiguity and complexity and greater openness to new experiences appear to be associated with liberal cognitive styles [6].

A useful distinction can be drawn between *social* and *economic* conservatism: individuals (and political parties) can be differentially placed on social and economic dimensions, such that it is possible to be economically conservative and socially liberal (as with some libertarians), or socially conservative and economically liberal (as with some populists). *Social*, or cultural, conservatism refers to the “preservation of ancient moral traditions of humanity” and includes the assumption that “political problems at bottom are religious and moral problems” ([13] – p.8). In contrast, *economic* conservatism refers to a dimension of attitudes that are concerned with the involvement of the government and the regulation of private enterprise in the economic lives of its citizens [14], [15].

Given the dynamic state of research in political psychology, it is important to have an appropriate scale to measure such differences in political attitudes on the left-right dimension. At present, however, there is a conspicuous lack of an appropriate scale that is concise, modern in its conceptualisation, accepted and validated, and that includes both social and economic conservatism. The scale proposed here is designed to fill this gap.

### Existing scales and their limitations

In contemporary political psychology, ‘conservatism’ is operationalized and measured in numerous ways: even a cursory examination of some recent literature in political psychology reveals diverse ways of measuring conservatism, raising, for researchers new to the field, several pragmatic difficulties in assessing which approach is best in any particular investigation. Such measures include requiring participants to rate how liberal or conservative they are on a single dimension (e.g. [1, 8]); using self-report on three separate liberal/conservative dimensions [16]; partisan identification [17], [18]; unstandardized partial versions of traditional conservatism scales (e.g. [2, 14, 19]); and using individual differences measures as a proxy for conservatism (e.g. [7, 20]). In addition to such pragmatic difficulties, this wide diversity within measurements of conservatism raises a strong conceptual problem: in measuring conservatism in such different ways, conservatism *as a concept* is operationalized and understood in different ways.

To pick just one example, in measuring conservatism as (strength of) partisan affiliation, it is implied that conservatism is primarily a matter of political social identification: one assesses how attached one is to the particular political group to which they belong. By contrast, asking about how liberal or conservative one is along a single dimension implies that one is primarily interested in the participants’ ideological stance, regardless of actual party affiliation or voting behaviour. In this section I shall discuss the most common ways that conservatism has been measured, highlighting both advantages and limitations of the different approaches. In doing so, the need for a new scale will become apparent.

### Traditional scales

Conservatism has traditionally been measured by scales such as those provided by Wilson and Patterson [21], Kirton [22], and Henningham [23]. As noted by Henningham [23], however, conservatism scales need a “use by” date: scales measuring conservatism and liberalism are products of their day and age. Indeed, this trend has been shown through recent decades in the history of political psychology: In 1978 Kirton [22] updated and revised Wilson and Patterson’s scale created in the 1960s [21], which was in turn revised and updated by Henningham in the 1990s [23]. Now, two decades on, it seems a new scale is required. Henningham’s scale includes items such as ‘voluntary euthanasia’, ‘death penalty’, ‘Bible truth’, ‘legalised prostitution’, ‘condom vending machines’, and ‘pre-marital virginity’ and it is not clear that such items are sufficiently representative of the kind of issues important to contemporary conservatives in the U.S – let alone issues important to contemporary conservatives in other parts of the world. Indeed, psychologists who choose to use a scale often adapt such traditional measures, and therefore omit items they perceive to not be relevant [2], [14], [19]. While this is necessary to tap the contemporary nature of conservative political ideology and is commendable for attempting to assess the degree to which individuals endorse conservative views, there are fundamental problems with such an approach. First, these partial and improvised scales are not empirically validated as scales, raising concerns about how appropriate it is to use these in psychological investigations – particularly when the main variable of interest is political conservatism. Moreover, the items that are chosen to be in that particular improvised scale are dependent upon the investigators’ own beliefs and attitudes about conservatism. While the items chosen by the researchers may indeed be those that best typify conservatism, this is often based solely on theoretical and not empirical considerations. Finally, when using these improvised scales it is often not specified which exact items were retained and which items removed, so that it is difficult to see precisely how conservatism was measured. It is of crucial importance in having a scale to measure conservative political ideology in individuals that one measures issues that are currently of relevance. While this means that such scales will inevitably have a ‘use-by’ date, it is preferable that one has a validated scale open to all, rather than requiring researchers desiring to tap actual conservative beliefs to pick and choose their own items from these scales – even if this scale is only valid for 10 years.

### ‘Ideological’ scales

A further approach to measuring conservatism has been to use scales that tap support of certain ideologies. For example, Jost and colleagues [24] developed their Fair Market Ideology (FMI) scale, consisting of 25 items measuring the extent to which individuals endorse support of a fair market as a legitimate and fair economic system. Jost et al’s scale is important because it assesses participants’ ideological views in a way that is not value-laden and does not include unwarranted inferences. Despite this, however, the FMI is limited because of its length and detail. First, by requiring participants to assess the fairness of 25 policies and situations, the FMI exerts a much higher time load on participants than a single-item measure and so is less likely to be included in often already lengthy surveys. Secondly, this scale may be unfairly skewed towards individuals who are educated in economic market systems and so are in some position to have an opinion about them. Individuals who have received less education or who are less interested in politics may not be able to engage fully with the questions, again limiting its use as a wide-scale measure.

### Single item scale

In recent years, perhaps due to the difficulty of finding a modern and short conservatism scale, conservatism has often been measured by single item self report measures, requiring participants to rate how conservative or liberal they are on a 1–7 (or 1–9) scale [1], [5], [6]. Such an item has an important advantage in being very short to administer, thus allowing researchers to include other lengthy scales in surveys. While important as a short and one-item measure of political conservatism, there are problems with the use of such a measure that indicates a need for a scale of social and economic conservatism.

A crucial issue in the use of single item scales (or, indeed, with two separate dimensions of social and economic conservatism) concerns whether participants can accurately place themselves on this dimension. Indeed, research suggests that individuals may in fact find it difficult to accurately place themselves on a self-report conservative-liberal dimension. It has been noted, for example, that while many Americans characterise themselves as conservative, they would be characterized as liberal based on their attitudes towards a range of issues such as poverty and education [25]. Similarly, while Americans have become more liberal on issues like gay marriage and immigration [26], they have also become more likely to identify as conservative [27]. Do people overestimate their political conservatism (or liberalism)? Recent compelling evidence suggests that they do [28]. Zell & Bernstein [28] had participants indicate their self-perceived political orientation on a single dimension in addition to completing an “objective” measure of political orientation based on work from the Pew Research Center, where participants rated their agreement to a number of statements about political issues such as “Gays and lesbians should be allowed to marry legally” (reversed) and “Poor people have become too dependent on government assistance programs” ([28] – p.6), and found that participants showed a significant bias towards perceiving themselves as more conservative than they were. Indeed, Zell and Bernstein specifically endorse the future use of objective measurements of political beliefs in research, concluding that “it is advised that future research use more objective measures of political orientation to cluster respondents into groups” ([28] – p.5).

A second important problem with the single self-report political orientation item is that it does not effectively differentiate between individuals’ socially conservative and economically conservative beliefs [29], [30]. It has been found that attitudes concerning social and cultural issues are factorially distinct from economic issues [31], and that such social and economic conservatism may have different psychological correlates [14]. Importantly, using a single item dimension is especially unsuited to summarising the preferences of self-identified moderates since such individuals may be cross-pressured between their views on social and economic issues [29]. As Treier & Hillygus note from their empirical investigation on this issue, while “political rhetoric is clearly organised by a single ideological dimension…the belief systems of the mass public are multidimensional” ([29] - p.680). There is increasing evidence that individuals with socially conservative views may be psychologically different from those who exhibit economically conservative views, thus necessitating nuance in measuring and operationalizing conservatism [14], [32]. In contrast, others note that individuals’ social and economic political attitudes are often correlated [33] and so combined conservatism scores are seen as being appropriate for certain circumstances [6]. Both combined and distinct measures, then, can be argued to be appropriate in different circumstances.

A third problem with the single item scale is that there are statistical problems of truncation of range when comparing the relationship between conservatism measured by a single item with scores from a multi-item scale: a lack of finding between variables may be influenced by the use of a single item in one case, and multiple averaged items in another. As such, to compare most effectively the relationship of conservatism with other psychological constructs it is important to use multi-item scales to avoid unfair bias due to problems of truncation.

Despite such potential problems, it remains clear that in certain situations, a single item measure of conservatism can be effective [9]. In other cases, asking participants to rate their conservatism on two separate dimensions of social and economic conservatism can suffice. However, a fundamental issue remains: researchers often opt to use multi-item scales of ideology, and at present researchers do use a variety of differing (and problematic) scales [2], [14], [19], necessitating a new multi-item scale of conservatism that addresses these limitations. A new multi-item scale is required not to replace the singe-item scale, but to complement it.

### Social dominance orientation and right wing authoritarianism

A final approach to measuring conservatism has been to assess conservatism indirectly, by using scales that do not directly measure political conservatism, but rather beliefs, cognitive styles, and dispositions from which conservatism can be inferred. A common example of this is Social Dominance Orientation (SDO) [34], which reflects an individual’s endorsement of intergroup hierarchies and inequalities and correlates strongly with political conservatism (e.g. [34, 35]). Given this strong correlation it is then possible to infer greater endorsement of conservative ideology. An important concern remains, however, that conservatism is still theoretically distinct from SDO. As reflecting individuals’ endorsement of intergroup hierarchies, social dominance is clearly related to conservatism. Importantly, however, SDO is not synonymous with conservative ideology for it remains possible in principle for one to be a ‘principled conservative’ who is opposed to equality based on beliefs in equity, fairness, and responsibility rather than prejudice. As such, one cannot take scores on SDO to represent political conservatism per se.

Another example of this approach can be seen in the use of Right Wing Authoritarianism (RWA) [36]. RWA is a personality and ideological variable that taps willingness to submit to authorities, support of social conventions and norms, and hostility towards those who do not adhere to such social norms, and has been consistently associated with conservatism. However, as with SDO, it is not always possible to correctly infer political conservatism from high RWA scores. At a practical level, the relationship between conservatism and authoritarianism depends on the context: an authoritarian in Cold War Russia was likely to support the Communist Party and so be anti-capitalist, while authoritarians in the U.S. at the same time were likely to be opponents of communism. Further, as Stenner [37] argues, authoritarianism and conservatism are distinct because authoritarianism focuses on aversion to difference across space (i.e. diversity of people and beliefs at the present time), while conservatism reflects aversion to difference over time (i.e. change). As such, there is no logical connection between the two, even if they often co-occur in practice.

In addition to such logical errors between inferring political conservatism from a measure that explicitly taps a construct related to – though distinct from – support of conservative policies and political parties, there are also broader philosophical and ethical issues. Measuring conservatism in this way is an example of what is known in philosophy as a “thick concept”: a concept that has a descriptive content but that also has a negative evaluative load. In measuring conservatism through measuring SDO and RWA, the concept (‘conservatism’) acquires an evaluative load, since both SDO and RWA are conceptualised as measures of discrimination (SDO), aggression, and faulty reasoning (RWA). As such, to define conservatism based on these negatively laden evaluative concepts is to exhibit a value judgement on the nature of conservatism. Despite difficulty in practice, it is often accepted that objective science should be as value-free as possible [38], such that our scientific theories and methods tap ‘objective’ concepts that are not unduly value-laden. It is important, then, that our methodological operationalization of conservatism should be as neutral and value-free as possible, through assessing participants’ support of ‘conservative issues’ and support for conservative political parties and leaders. Conservatism in this operationalization may be associated with RWA and SDO, but that association is a further empirical step rather than one that adds value to the concept.

### Key requirements

It is clear, then, that there is sufficient ambiguity and concern about existing measures of conservatism to demonstrate the importance of a new contemporary scale of conservatism that addresses such limitations. Reflection on how traditional scales of conservatism are used shows that an important condition of a new scale is that it represents *contemporary* political conservatism, with clear and specified items that have been shown to be representative of conservatism. After discussion of the use of long ideology scales, it is suggested that the new scale should follow from such ideological measures in tapping participants’ ideological beliefs, though in a shorter and more accessible form. Based on the distinction between social and economic conservatism, it is important to provide a measure that recognises the independence of social and economic conservatism, allowing researchers to examine the psychological effects of economic and social conservatism in isolation, while also recognising that in practice these are often correlated and that composite measures can also be appropriate in other circumstances. Given that individuals can struggle to place themselves accurately on a single item dimension of conservatism, a further requirement is that the measure should be relatively easy for participants to respond to accurately. Reflection on the problem of truncation revealed that the new scale should have multiple items to improve reliability. Finally, consideration of the use of RWA and SDO as proxies for conservatism led to a final requirement that a new scale avoids evaluative connotations and assesses conservative political belief as objectively as possible. In summary, then, several requirements were considered in the development of the present scale:

1. It should reflect the nature of contemporary conservatism.
2. It should be short enough to facilitate easy administration.
3. It should not require ‘specialist’ knowledge on the details of specific policies.
4. It should consist of multiple items to avoid problems of truncation.
5. It should include items measuring both economic and social conservatism.
6. It should provide a value-free representation of the extent to which individuals exhibit support of issues and values characteristic of conservatism.

Based on these requirements, in this paper a new measure of political conservatism is proposed and validated: the 12-item Social and Economic Conservatism Scale (SECS). This scale is explicitly designed to measure what has been referred to as “peripheral” aspects of conservatism: “attitudes concerning the size of government, military spending, or immigration policies that vary in their ideological relevance across time and place” ([9] -p.654). While disagreement may occur as to whether the actual content of conservative beliefs should be seen as peripheral in understanding conservatism, in this article I accept and endorse this broad distinction. The SECS is designed to measure individuals’ support for the so-called ‘peripheral’ aspects of conservatism: conservatism as a general approach to political issues, regardless of actual party affiliation or underlying personality type. In this sense, the SECS is not in opposition to scales measuring party affiliation, perceived self ideology, or the ‘core’ underlying personality traits of conservatives, but rather complementary.

## Methods

### Ethics statement

Relevant ethical guidelines were followed and the research was approved through University of Oxford’s Central University Research Ethics Committee, with the reference number MSD-IDREC-C1-2012-161. Written informed consent was obtained electronically for all participants.

### Item selection

To ensure that the SECS assessed the contemporary nature of conservatism (vs. liberalism), an exploratory study was first conducted to investigate what laypeople consider characteristic of political conservatism. Forty-one American participants (13 female) participated online using Amazon Mechanical Turk (MTurk), and were paid $0.50 for their time. MTurk is a website that facilitates payment for completing tasks posted by researchers, and such samples have been shown to provide reliable data and be more representative of the general population than student samples [39]. Only American participants were able to take part in the study, all participants completed the survey fully, and payment was facilitated via the Amazon website. The mean age of participants was 32 years old (*SD*  =  10.19), and participants were predominately white (*N* = 35).

Participants were asked to “Please write down what you think are the 10 main issues that are important in characterising political conservatism. For example, you may think that traditional values characterises contemporary conservatism”. Results revealed that abortion, small government, welfare benefits, low taxes, the military, religion, gun ownership, traditional marriage, immigration, traditional values, fiscal responsibility, business, the family unit, patriotism, capitalism, climate change, the death penalty, personal responsibility, strict laws, evolution, and education were all issues that participants judged as being important in characterising political conservatism (see Materials S2). It is noteworthy that many items from traditional scales of conservatism, such as Wilson and Patterson’s [21] and Henningham’s [23] were not listed as being important in characterizing contemporary political conservatism, thus providing strong evidence for the need of an updated scale of conservatism. For scale development, items that were reported by at least 15% of participants were included, meaning that items 15 to 22 were excluded. The importance of these issues to conservatism was then confirmed through examination of other theoretical accounts of conservatism (e.g. [29]).

### Participants and procedure

To validate the scale, 319 American participants were recruited again via MTurk, and completed the survey online. To ensure the quality of the data, participants were excluded from further analysis if they completed the survey in too short a time or did not pass an attention check (<250sec). This cut-off point represented the minimum realistic time that a participant could have completed the survey reading every question, and the attention check constituted an item embedded in the survey where participants were told to “Please enter scale point -4 to confirm you are paying attention”. As such, the final number of participants included in data analysis was 291 (126 females). The mean age of participants was 37 years old (*SD*  =  13.28), and participants were predominantly White (*N* = 248). A range of political party identification was found, with all main American parties (or positions) represented: Republican (*N* = 49), Democrat (*N* = 134), Independent (*N* = 90), and the remainder selecting a party other than these (*N* = 30).

### SECS scale

Participants were then given a list of 14 words or phrases representing issues important to conservatism and asked to rate them on a commonly used ‘feeling thermometer’ - “How positive or negative do you feel about each issue on the scale of 0 to 100, where 0 represents very negative, and 100 represents very positive?” Such 0-100 thermometers have been consistently and reliably used throughout social psychology, allowing participants to express the strength of their feeling, or indeed their neutral feelings if they choose the mid-point [40]. This use of warmth ratings has been well implemented in previous work from social psychology [41], and allowed individuals to express their position towards the items while not requiring them to have any specific knowledge about policies. Following from Henningham’s scale, the use of words or phrases instead of specific statements was chosen to allow responses indicative of participants’ responses to the issue in general, rather than any specific attitudes concerning distinct policies. Despite being (purposely) unspecified, results revealed remarkable consensus in understanding of these terms, highlighting that even certain issues (as opposed to specific policies) are reliably and consistently seen as being representative of conservatism. Scores were ‘tied’ to multiples of 10, such that participants could respond with 10, 20, 30, and so on. This was chosen to allow participants to respond in a way that allowed range in their responses (unlike Henningham’s bipolar response scale) and to not require knowledge or opinion on specific policies or laws. The order of items was randomised for each participant to control for potential order effects. The 14 items presented (in this wording) were:

1. Abortion.
2. Welfare benefits (reverse scored).
3. Tax (reverse scored).
4. Immigration (reverse scored).
5. Limited government.
6. Military and national security.
7. Religion.
8. Gun ownership.
9. Traditional marriage.
10. Traditional values.
11. Fiscal responsibility.
12. Business.
13. The family unit.
14. Patriotism.

## Results

### Items excluded

In analysing results, I first examined the correlation matrix to ensure that all items were consistently associated with each other. Given that a key parameter for this scale was that it should be comparatively short (and therefore easily administered), a decision was made to try and limit items included in the final scale to 12. Based on low relative inter-correlations with other items, two items were strong candidates for exclusion: immigration and tax. In addition to practical reasons for exclusion, these two items were also flagged as being potentially difficult due to their ambiguity theoretically. While self-reported conservatism was associated in the predicted directions with these two items, there was still concern that both items were still too ambiguous and not representative of contemporary conservative thought in America. Immigration of skilled workers, for example, is often lauded as a positive aspect of the free market, while illegal immigration and immigration for low paid jobs are seen as more problematic. With regards to tax, concern existed that this is importantly linked to the degree, spread, and type of tax, which may introduce confounding factors. Given all these considerations, the two items of immigration and tax were excluded from the scale and further factor analyses were conducted solely with the twelve items retained.

The twelve items retained can be seen to be representative of American conservatism in particular, given items such as ‘gun ownership’. While this is accepted as a limitation for its utility for cross-cultural research, having a specific, clearly-defined, and value-free measure of the peripheral aspects of conservatism is crucial to conducting research in political psychology, and the vast majority of research takes place in the U.S. While this 12-item SECS scale is unlikely to accurately measure, for example, sub-Saharan African conservatism, it can be argued that the benefits of having a scale that addresses the limitations revealed by the literature review outweigh this.

### Exploratory factor analysis

Next, exploratory factor analysis (EFA) was conducted, following the ‘best practices’ recommended in the literature [42], [43], [44], [45]. Guided by theoretical considerations and advice on best practices in exploratory factor analysis, *principal axis factors* extraction was used over the more common principal components analysis [42], [45]. Principal components analysis is primarily a data reduction method, computed without regard to the structure caused by latent variables [46]. In contrast, factor analysis reveals latent variables that cause variables to covary, and so is more appropriate for scale development [42]. The ‘oblique’ direct oblimin rotation was chosen as factors were expected to correlate in measuring facets of conservatism, and using an orthogonal (e.g. Varimax) method can result in loss of valuable information when factors are correlated [44].

In this EFA, the factorability of the 12 items was first examined. Tests for multicollinearity revealed a low level of multicollinearity (*VIF*  = .004) (See Table 1 for correlation matrix). The KMO measure of sampling adequacy was .88, above the recommended value of .6, and Bartlett’s test of sphericity was significant: χ^2^ (66)  = 1542.04, *p*<.001. Given these overall indicators, factor analysis was conducted with all 12 items. Principal axis factoring extraction using direct oblimin rotation was used, and three factors with eigenvalues greater than 1 were extracted. The first factor explained 43% of the variance (*eigenvalue*  =  5.21), the second factor 13% of the variance (*eigenvalue*  =  1.60), and the third factor 8% of the variance (*eigenvalue*  =  1.01).

**Table 1:** Correlation Matrix for SECS Items.

	1	2	3	4	5	6	7	8	9	10	11	12	13
1. Abortion													
2. Religion	.55^**^												
3. Gun Ownership	.26^**^	.25^**^											
4. Traditional Marriage	.52^**^	.58^**^	.32^**^										
5. Traditional Values	.62^**^	.66^**^	.39^**^	.78^**^									
6. The Family Unit	.43^**^	.43^**^	.33^**^	.61^**^	.64^**^								
7. Patriotism	.40^**^	.46^**^	.36^**^	.52^**^	.55^**^	.54^**^							
8. Military and National Security	.29^**^	.39^**^	.27^**^	.39^**^	.44^**^	.38^**^	.63^**^						
9. Limited Government	.23^**^	.09	.54^**^	.24^**^	.27^**^	.32^**^	.24^**^	.17^**^					
10. Fiscal Responsibility	.19^**^	.09	.31^**^	.22^**^	.26^**^	.30^**^	.37^**^	.31^**^	.42^**^				
11. Business	.27^**^	.29^**^	.39^**^	.35^**^	.43^**^	.39^**^	.42^**^	.42^**^	.37^**^	.40^**^			
12. Welfare Benefits	.27^**^	.24^**^	.43^**^	.25^**^	.35^**^	.21^**^	.34^**^	.20^**^	.44^**^	.25^**^	.36^**^		
13. Tax* (Excluded)	.28^**^	.18^**^	.20^**^	.28^**^	.26^**^	.27^**^	.22^**^	.03	.39^**^	.15^**^	.14^*^	.46^**^	
14. Immigration* (Excluded)	.24^**^	.19^**^	.31^**^	.34^**^	.36^**^	.26^**^	.35^**^	.22^**^	.29^**^	.19^**^	.19^**^	.43^**^	.35^**^

**Note**: * indicates that the correlation is significant at the *p*<.05 level; ** indicates that the correlation is significant at the *p*<.01 level.

Using the ‘scree test’ [47], the scree plot revealed a break in the eigenvalues after the second factor, indicating that a two-factor solution should be adopted. Further, the third factor had only two items, and it is recommended that factors with fewer than three items should not be retained [48]. Interpretability of the first two factors was high: social issues loaded primarily onto the first factor, while economic issues loaded primarily on the second factor, and cross-loadings between factors were low (see Tables 2 and 3). Given that the two-factor solution appeared to best represent the data, a second principal axis factoring extraction using direct oblimin rotation was used, this time with a specification to extract two factors (see Table 4 for primary factor loadings). In this two-factor solution, no variables cross-loaded (i.e. were greater than .32: [48]), and all variables had moderate to good loadings, with a minimum loading of .45 [44]. Reliability analyses confirmed internal consistency, with a good overall Cronbach’s alpha of .88 for the complete 12-item scale, an alpha of .70 for the 5-item economic conservatism subscale, and an alpha of .87 for the 7-item social conservatism subscale. As such, exploratory factor analysis supported the development of this 12-item scale of Social and Economic Conservatism (SECS), with items loading strongly onto either the social or economic factor, and good overall consistency for the complete 12-item scale (See Materials S1).

**Table 2:** Factor Matrix using Principal Axis Factoring (PFA) and Direct Oblimin Rotation.

	Factor		
	1	2	3
Limited Government	.49	.63	–.23
Military and National Security	.60	–.05	.47
Religion	.64	–.37	–.10
Gun ownership	.55	.38	–.12
Traditional marriage	.76	–.28	–.14
Traditional Values	.86	–.29	–.20
Fiscal responsibility	.44	.34	.16
Business	.58	.23	.13
The family unit	.69	–.09	–.01
Patriotism	.74	–.04	.36
Abortion	.62	–.21	–.19
Welfare Benefits	.47	.30	–.12

**Table 3:** Pattern Matrix using Principal Axis Factoring (PFA) and Direct Oblimin Rotation.

	Factor		
	1	2	3
Abortion	.68		
The family unit	.50		
Religion	.75		
Traditional marriage	.79		
Traditional Values	.90		
Fiscal responsibility		.41	
Business		.36	
Limited Government		.89	
Gun ownership		.61	
Welfare Benefits		.51	
Patriotism			.65
Military and National Security			.75

**Table 4:** Pattern Matrix using Principal Axis Factoring (PFA) to extract two factors with Direct Oblimin Rotation.

	Factor	
	1	2
	“Social Conservatism”	“Economic Conservatism”
Abortion	.65	
The family unit	.60	
Religion	.82	
Traditional marriage	.83	
Traditional Values	.90	
Patriotism	.56	
Military and National Security	.45	
Fiscal responsibility		.54
Business		.49
Limited Government		.81
Gun ownership		.64
Welfare Benefits		.52

### Confirmatory factor analysis

Next, a confirmatory factor analysis (CFA) was performed to assess the factor structure of the SECS revealed by the EFA. The CFA was conducted using the programme MPlus (Version 6.1) [49], and items were allowed to covary. All the items fell well within the appropriate skewness (±2) and kurtosis (±7) values recommended by West, Finch, and Curran [50], and so are sufficiently normal (See Table 5). The measurement model fit the data well, **χ^2^** (52)  = 129.94, *p* <.001, **χ^2^** / *df* = 2.50. The chi-square statistic tests the null hypothesis that there are no differences between the observed and model-implied covariance matrices [51]. Although the chi-squared values were significant (suggesting potential poor fit) this is because type I error is extremely sensitive to sample size, which can increase artificially the chi-squared values. Accordingly, criticisms have been levelled at the chi-square test as an appropriate test of good model fit [52], [53], despite continued recommendation to include it in reports. As such, Hu and Bentler [51] recommend including at least two other fit statistics. In line with this, the chi-square ratio was also included, where a ratio between 2 and 3 is considered acceptable fit [54]. The chi-square ration for this CFA was 2.50, thus indicating good fit. In addition, the standardized root mean square residual (SRMR) was inspected. The SRMR ranges between 0 and 1 with values closer to zero indicating better fit, and SRMR ≤.08 being indicative of an acceptable model fit [51]. The SRMR for this CFA was .06, thus representing a good fit for the model. Finally, the root mean square error of approximation (RMSEA) was included, where values closer to 0 indicate good fit, and a RMSEA of ≤ .06 indicating good fit. In this CFA, the RMSEA was .07, which while not at the optimal limits recommended by Hu & Bentler [51], was well within the boundaries of acceptable fit. As such, CFA confirmed that the model outlined in the EFA of a two-factor structure of the SECS was an appropriate fit, justifying its use as a scale.

**Table 5:** Skewness and Kurtosis of the 12 SECS Items.

	Skewness	Kurtosis
Abortion	0.24	–1.35
Welfare Benefits	0.43	–0.69
Limited Government	–0.31	–1.05
Military and National Security	–0.28	–0.97
Religion	0.55	–1.23
Gun Ownership	0.07	–1.46
Traditional Marriage	0.14	–1.41
Traditional Values	0.36	–1.25
Fiscal Responsibility	–0.86	0.30
Business	–0.48	–0.50
The family unit	0.76	–0.37
Patriotism	–0.28	–1.18

### Relationships with established measures

Descriptive statistics revealed that overall SECS scores were around the 5.0 midpoint of the scale (*M* = 5.80, *SD*  =  1.94). It is notable that mean scores on the SECS were slightly above the scale midpoint, while mean self-reported overall conservatism (measured by the single item measure) were almost a whole scale point below the scale midpoint of 4.00 (*M* = 3.14, *SD*  =  1.71).

Finally, construct validity was assessed through correlational analyses that were conducted to see how well scores on the SECS correlated with other constructs that one should expect for a scale measuring social and economic conservatism (See Table 6). Further, linear regression analyses were then conducted to investigate how well self-reported ideology and SECS scores predicted scores on these other constructs. These analyses were conducted independently in separate linear regressions, due to the high correlation between self reported ideology and SECS scores.

**Table 6:** Correlation Matrix to show the relationships between scores on SECS and other measures.

	1	2	3	4	5	6	7	8	9
1. SECS									
2. SECS Social Conservatism	.96^**^								
3. SECS Economic Conservatism	.75^**^	.55^**^							
4. RWA	.76^**^	.77^**^	.52^**^						
5. SDO	.49^**^	.39^**^	.56^**^	.54^**^					
6. Dogmatism	.42^**^	.44^**^	.24^**^	.56^**^	.27^**^				
7. System Justification	.40^**^	.38^**^	.29^**^	.32^**^	.21^**^	.16^**^			
8. Fair Market	.58^**^	.47^**^	.64^**^	.47^**^	.49^**^	.21^**^	.60^**^		
9. Resistance to Change	.38^**^	.36^**^	.31^**^	.41^**^	.37^**^	.20^**^	.52^**^	.46^**^	
10. Self-Report Conservatism	.71^**^	.65^**^	.62^**^	.72^**^	.59^**^	.39^**^	.29^**^	.53^**^	.39^**^

**Note**: * indicates that the correlation is significant at the *p*<.05 level; ** indicates that the correlation is significant at the *p*<.01 level.

### Relationship to political affiliation and reported ideology

First, it was found that overall conservatism measured by the SECS correlated significantly with the measure of self-reported conservatism on a 1–9 scale (*r* = .71, *p*<.001). Next, analyses were conducted to investigate whether there were significant differences on scores on the SECS as a function of political party affiliation (Democrat, 134; Republican, 49). Independent samples t-test revealed significant differences on overall SECS scores, *t*(181)  = 13.30, *p*<.001, with higher scores for Republicans (*M* = 7.96) than Democrats (*M* = 4.89). Further, there were significant differences on economic conservatism SECS scores, *t*(181)  = 11.44, *p*<.001, with higher scores for Republicans (*M* = 7.91) than Democrats (*M* = 5.39). Similarly, there was a significant difference for social conservatism, *t*(181)  = 10.41, *p*<.001, with higher scores on the SECS for Republicans (*M* = 8.14) than Democrats (*M* = 4.66). In tapping differences in political beliefs on a liberal-conservative spectrum, then, the SECS works well to highlight these differences.

### Relationship to social dominance, system justification, and resistance to change

Jost and colleagues [6] have proposed an influential *motivated social cognition* framework to explain individual differences in political ideology, suggesting that those who embrace a right wing ideology—characterized by resistance to change and acceptance of traditional hierarchies — may do so in part because it serves to reduce certain motivational needs. Further, research has also demonstrated that liberals exhibit stronger implicit as well as explicit preferences for social change and equality when compared with conservatives, as well as reduced system justification (e.g. [6, 55]). As such, I tested the extent to which SECS scores were associated with scores on established measures of resistance to change [56], system justification [55], and social dominance orientation [34].

SDO was measured in a scale taken from Pratto and colleagues [34] and consisted of eight items to which participants indicated how much they agreed or disagreed on a 7-point Likert scale. Items included ‘It is probably a good thing that certain groups are at the top and other groups are at the bottom’ and ‘We should do what we can to equalize conditions for different groups’ (reversed) (Cronbach’s α = .92). Overall SECS scores were significantly correlated with SDO (*r* = .49, *p*<.001), as were scores on both the economic (*r* = .56, *p*<.001) and social conservatism subscales (*r* = .39, *p*<.001). Self-reported ideology explained 35% of the variance in social dominance, *R* ^2^ = .35, *F* (1,289)  = 152.09, *p*<.001, while SECS scores explained a smaller 24% of the variance, *R* ^2^ = .24, *F* (1,289)  = 89.57, *p*<.001.

Resistance to change was measured through two items taken from the work of Jost and colleagues [56], consisting of two items on a Likert scale to which participants rated how much they agreed (7) or disagreed (1). The two items were ‘I would be reluctant to make any large-scale changes to the social order’ and ‘I have a preference for maintaining stability in society, even if there seem to be problems with the current system’. (Cronbach’s α = .74; *r* = .59, *p*<.001). Overall SECS scores were significantly correlated with resistance to change (*r* = .38, *p*<.001), as were scores on both the economic (*r* = .31, *p*<.001) and social conservatism subscales (*r* = .36, *p*<.001). Self-reported ideology explained 15% of the variance in resistance to change, *R* ^2^ = .15, *F* (1,279)  = 48.98, *p*<.001, while SECS scores explained 14% of the variance in resistance to change, *R* ^2^ = .14, *F* (1,279)  = 46.50, *p*<.001.

System justification was measured in an 8-item scale developed by Kay and Jost [55]. Participants indicated how much they agreed or disagreed with a number of statements on a 1 (strongly agree) to 9 (strongly disagree) scale, including “In general you find society to be fair”, and “American society needs to be radically restructured” (reversed). (Cronbach’s α = .83). Overall SECS scores were significantly correlated with system justification (*r* = .42, *p*<.001), as were scores on both the social (*r* = .38, *p*<.001) and economic conservatism subscales (*r* = .29, *p*<.001). Self-reported ideology explained 9% of the variance in system justification, *R* ^2^ = .09, *F* (1,289)  = 27.32, *p*<.001, while SECS scores explained 16% of the variance in system justification, *R* ^2^ = .16, *F* (1,289)  = 56.17, *p*<.001.

### Relationship to RWA

Right Wing Authoritarianism (RWA) was measured using Zakrisson’s [57] short version of the RWA scale, consisting of 15 items including “God’s laws about abortion, pornography and marriage must be strictly followed before it is too late, violations must be punished” (Cronbach’s α = .93). Overall SECS scores were significantly correlated with RWA (*r* = .76, *p*<.001), as were scores on both the social (*r* = .77, *p*<.001) and economic conservatism subscales (*r* = .52, *p*<.001). Self-reported ideology explained 52% of the variance in RWA, *R* ^2^ = .52, *F* (1,289) = 307.31, *p*<.001, while SECS scores explained a similarly sized 58% of the variance in RWA, *R* ^2^ = .58, *F* (1,289)  = 405.74, *p*<.001.

### Prejudice

Prejudice was measured using a widely used ‘feeling thermometer’, where participants were asked to rate how warm or cold they felt about four out-groups (feminists, homosexuals, welfare recipients, and the homeless) on a scale of 0 to 100 (Cronbach’s α = .71). Overall SECS scores were significantly correlated with prejudice (*r* = –.45, *p*<.001), as were scores on both the social (*r* = –.39, *p*<.001) and economic conservatism subscales (*r* = –.51, *p*<.001). Self-reported ideology explained 37% of the variance in prejudice, *R* ^2^ = .37, *F* (1,289)  = 168.19, *p*<.001, while SECS scores explained a lower 21% of the variance in prejudice *R* ^2^ = .21, *F* (1,289)  = 75.07, *p*<.001. As such, self-reported ideology seemed to be associated more strongly with prejudice than SECS scores, highlighting the importance of choosing one’s measures carefully.

### Dogmatism

Dogmatism was measured using Altemeyer’s [58] 20 item scale, including items such as “I am absolutely certain that my ideas about the fundamental issues in life are correct”, to which participants indicated how much they agreed or disagreed on a -4 (strongly disagree) to +4 (strongly agree) scale (Cronbach’s α = .89). Overall SECS scores were significantly correlated with dogmatism (*r* = .42, *p*<.001), as were scores on both the social (*r* = .44, *p*<.001) and economic conservatism subscales (*r* = .24, *p*<.001). Self-reported ideology explained 15% of the variance in dogmatism, *R* ^2^ = .15, *F* (1,289)  = 50.47, *p*<.001, while SECS scores similarly explained 18% of the variance in dogmatism, *R* ^2^ = .18, *F* (1,289)  = 62.98, *p*<.001. As such, both self-reported ideology and SECS explained similar amounts of variance in dogmatism.

### Relationship to fair market ideology

To investigate how well scores on the SECS – and particularly on the economic conservatism sub-scale – correlated with fair market ideology, participants also completed Jost, Blount, Pfeffer, and Hunyady’s [24] Fair Market Ideology scale. Belief in a fair market is an important part of economic conservatism, and so scores on SECS should be expected to correlate with fair market ideology. The scale consisted of 25 items in total, to which participants rated how much they agreed with 15 statements on a -5 (completely disagree) to +5 scale (completely agree), including “The free market system is a fair system”. Next, participants indicated how fair they thought a number of scenarios were on a -5 (completely unfair) to +5 scale (completely fair) scale, such as “The fact that wealthier people live in bigger homes and better neighborhoods than poorer people who cannot afford to pay the same prices is…” (Cronbach’s α = .93). Overall SECS scores were significantly correlated with fair market ideology (*r* = .58, *p*<.001), as were scores on both the social (*r* = .47, *p*<.001) and economic conservatism subscales (*r* = .64, *p*<.001). Self-reported ideology explained 28% of the variance in fair market ideology, *R* ^2^ = .28, *F* (1,289)  = 113.82, *p*<.001, while SECS scores explained a larger 34% of the variance in fair market ideology, *R* ^2^ = .34, *F* (1,289)  = 147.47, *p*<.001.

## Discussion

An essential prerequisite of conducting good psychological research is to have a good methodology, of which measurement of one’s target construct is a fundamental part. In this paper limitations of previous approaches to measuring conservatism have been discussed, showing that there is a strong theoretical and pragmatic need for a new multi-item scale of conservatism. In particular, based on this evaluation of existing measures, seven ‘key requirements’ that were necessary for the proposed scale to meet to improve upon previous measures were formulated. These requirements were that:

1. It should reflect the nature of contemporary conservatism.
2. It should be short enough to facilitate easy administration.
3. It should not require ‘specialist’ knowledge on the details of specific policies.
4. It should consist of multiple items to avoid problems of truncation.
5. It should include items measuring both economic and social conservatism.
6. It should provide a neutral value-free representation of the extent to which individuals exhibit support of issues and values characteristic of conservatism.

Based on these requirements, a new scale for measuring political conservatism has been constructed. Based on empirical and theoretical considerations, 12 items shown to be important in representing both economic and social conservatism constituted the scale. Items assessing conservative views on both social and economic issues are included, and in line with previous research these are correlated though distinct [14], [31].

As found by Zell & Bernstein [28], there was discrepancy between how participants placed themselves on the single item dimension vs. their scores on the SECS. In particular, in this study participants tended to rate themselves as more liberal on the single-item identification scale than exhibited on the SECS scale. Analyses revealed that scores on the SECS were strongly associated with self-reported ideology and political affiliation and a variety of individual differences variables associated with conservatism, thus confirming its validity. Further, such analyses revealed both similarity and disparity in the relationship of SECS and self-reported ideology scores with other constructs, with SECS scores showing a tendency to be more related to actual conservative political beliefs, with self-reported ideology tending to be more associated with negative intergroup attitudes. Such findings confirm a core tenet of the argument presented here, and so provide yet more justification for this scale: how conservatism is measured is fundamental to looking at the relationship it has to other variables.

### Limitations and caveats

The SECS, then, has been demonstrated to be a valid measure tapping support for conservative ideological positions, thus providing an accurate way of assessing an individual’s ‘peripheral’ conservatism to be used in addition to those tapping the ‘core’ underlying beliefs and attitudes of conservatives. However, as with all the measures of conservatism reviewed, there are limitations of this measure that it is necessary to make explicit.

First, the SECS does not – and indeed could not – provide an inherent remedy to the issue of socio-cultural time dependence. In focusing on the peripheral aspects of conservatism, this measure has been explicitly designed to measure “peripheral issues (such as attitudes concerning the size of government, military spending, or immigration policies) that vary in their ideological relevance across time and place” ([9] - p.654). As such, the version of the SECS presented here is unabashedly a scale that will at some point become outdated, for it is impossible to tap participants’ conservatism relating to specific political issues without tying the scale to a certain context. The SECS, then, is likely to require updating in 10 years’ time. As argued before, however, the benefits of having a valid, standardised, and accurate measure of assessing conservative political preferences outweighs the limitation of having to update the scale at some point in the future. Given that researchers at present still opt to use multi-item scales of the peripheral aspects of conservatism [2], [14], [19], there is a demonstrable need for a standard scale: even if this scale, like its predecessors, has a ‘use-by’ date.

Secondly, this scale was explicitly designed as a measure of political conservatism, given that much research highlights and focuses on conservatism as the referent object primarily to be explained. Of course, as Jost and others have argued (e.g. [59]), the liberal-conservative dimension does seem to be effective in characterising political positions, and so it is likely that the SECS will help to identify liberal political views as well as conservative political views. However, it is important to note that this scale was explicitly designed – as its name indicates – to provide primarily a measure of political conservatism, and so the extent to which this scale is wholly effective in research questions centred around political liberalism has not yet been determined.

Finally, it is important to reiterate again that in this paper avoidance of the different measurements of conservatism discussed is not recommended. While there are crucial limitations of the different methods that demonstrate the need for a new multi-item scale, the use of a particular method must depend upon its suitability for the research question being addressed. In particular, the single-item measure of conservatism showed good predictive power alongside the SECS, and so this study indirectly provides further support for this measure’s validity. Given this, I note that in some circumstances the single-item measure (or indeed other measures reviewed here) may be most appropriate, while simultaneously maintaining that the SECS may be more effective in other circumstances and provides a good multi-item measure of conservatism for researchers who would prefer not to use a single item scale. In evaluating and addressing the respective strengths and weaknesses of different measurements of conservatism, this paper demonstrates the importance of choosing scientifically appropriate measures for the specific question to be addressed.

## Conclusions

To conclude, in this paper I have shown the SECS to be a useful addition to measurements of political ideology. I have demonstrated that the SECS provides a good alternative to (though not a replacement of) existing measures of conservatism such as out-dated scales, long ideology scales, single item measures, or the use of inferring conservatism from individual differences measures. To understand the psychology of political ideology, one must measure ideology in the most appropriate way. It is for this reason that the SECS scale is proposed, and for this reason that the SECS is suggested to be an important and valuable addition to the political psychologist’s toolbox.

## Supporting Information

Materials S1 **12 Item SECS Scale.**
(DOCX)

Materials S2 **Pilot Study Results.**
(DOCX)
