# Supplementary material for: The 12 Item Social and Economic Conservatism Scale (SECS)
Source: PLoS One. 2013 Dec 11;8(12):e82131. doi: 10.1371/journal.pone.0082131 (PMC3859575; doi:10.1371/journal.pone.0082131)
Supplement: Materials S1 — 12 Item SECS Scale. (DOCX) [file pone.0082131.s001.docx]

**Supplementary Materials 1: The 12 Item Social and Economic Conservatism Scale (SECS)**

“Please indicate the extent to which you feel positive or negative towards each issue. Scores of 0 indicate greater negativity, and scores of 100 indicate greater positivity. Scores of 50 indicate that you feel neutral about the issue.”

1. Abortion (reverse scored). (S)
2. Limited government. (E)
3. Military and national security. (S)
4. Religion. (S)
5. Welfare benefits (reverse scored). (E)
6. Gun ownership. (E)
7. Traditional marriage. (S)
8. Traditional values. (S)
9. Fiscal responsibility. (E)
10. Business. (E)
11. The family unit. (S)
12. Patriotism. (S)
