# Supplementary material for: The 12 Item Social and Economic Conservatism Scale (SECS)
Source: PLoS One. 2013 Dec 11;8(12):e82131. doi: 10.1371/journal.pone.0082131 (PMC3859575; doi:10.1371/journal.pone.0082131)
Supplement: Materials S2 — Pilot Study Results. (DOCX) [file pone.0082131.s002.docx]

**Supplementary Materials 2: Pilot Study Results**

Results from the Pilot Study detailing contemporary issues that characterize conservatism (% of participants indicating this item shown in brackets)

1. Anti-abortion (55%)
2. Less Government (55%)
3. Reduced Welfare (55%)
4. Low taxes (50%)
5. Military (50%)
6. Religion (45%)
7. Guns (39%)
8. Traditional marriage (33%)
9. Immigration (33%)
10. Traditional Values (31%)
11. Fiscal responsibility (31%)
12. Business (29%)
13. Family (19%)
14. Patriotism (19%)
15. Capitalism (14%)
16. Climate Change (7%)
17. Death Penalty (7%)
18. Personal Responsibility (5%)
19. Laws (5%)
20. Evolution (2%)
21. Education (2%)
